# Variation analysis using random forests reveals domestication patterns and breeding trends in sugar beet

**DOI:** 10.1016/j.isci.2025.112835

**Published:** 2025-06-11

**Authors:** Felix L. Sandell, Christina Rupprecht, Heinz Himmelbauer, Juliane C. Dohm

**Affiliations:** 1 Institute of Computational Biology, Department of Biotechnology and Food Science, BOKU University, Muthgasse 18, 1190 Vienna, Austria; 2 Institute of Integrative Nature Conservation Research, Department of Ecosystem Management, Climate and Biodiversity, BOKU University, Gregor-Mendel-Straße 33, 1180 Vienna, Austria

**Keywords:** Plant genetics, Molecular genetics, Microbial genetics, Plant biology, Agricultural science

## Abstract

Cultivated beets (*Beta vulgaris*), including sugar beet, are important crops, and several studies employed whole genome sequencing to explore genomic variation. We applied the machine learning method “random forests” on hundreds of sequenced beet accessions and identified genomic variants that distinguish wild from domesticated beets at a mean accuracy of 98.4%. Associated genes were involved in sugar accumulation and transport (e.g., SUC4), nematode resistance, and root growth. Modern breeding lines from leading seed companies were distinguished from public seed bank accessions at 98.5% accuracy, revealing a strong signal linked to fungal resistance, likely originating from Italian wild beets. We also differentiated accessions by company, uncovering genes under selection, notably the flowering regulator APETALA1. Admixture profiles were analyzed to address open questions regarding the genomic history, provenance, and dispersal of wild beets. Our findings provide exciting possibilities for targeted breeding and show advances in variation analysis using machine learning.

## Highlights

• Machine learning successfully identifies key genomic variation in beets
• Domestication genes linked to sugar transport, root growth, and nematode resistance
• A likely *Erysiphe* resistance is distinctive of modern sugar beet breeding lines
• Admixture profiles reveal history, provenance, and dispersal of wild beets

## Graphical abstract

**Figure:**
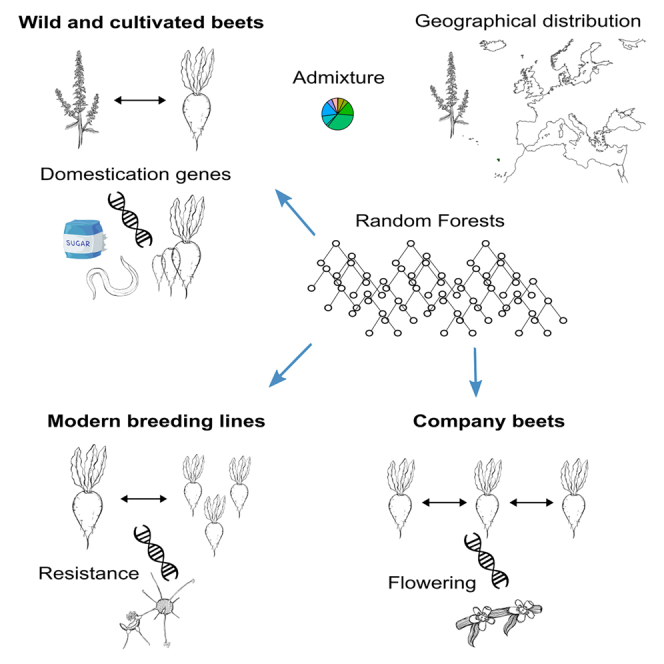

## Teaser

Plant genetics; Molecular genetics; Plant biology; Agricultural science; Machine learning

## Introduction

Sugar beet, a member of the Amaranthaceae family (order Caryophyllales) which also includes quinoa, amaranth, and spinach, is a highly important crop worldwide. In 2022, the global sugar beet harvest exceeded 260 million metric tons.^1^ Beets of the genus *Beta*, section *Beta* are diploid species with 2*n* = 18 chromosomes and encompass the cultivated variations sugar beet, table beet, leaf beet (chard), as well as the wild beets *Beta vulgaris* ssp. *maritima* (sea beet), *B. vulgaris* ssp. a*danensis*, *B. macrocarpa*, and *B. patula*.^2^ These species and subspecies can readily crossbreed, making the wild members of the genus a valuable gene pool for crop improvement. Many beneficial traits present in the wild relatives have been lost during domestication, rendering them important targets for commercial plant breeders.^3^

Sea beets are predominantly found in coastal regions, forming a belt along the upper high tide level. Inland populations are rare and associated with man-made habitats and human disturbances.^4^ The species' distribution has been shaped by historical events, including the last glaciation, during which the distribution of sea beets along the Atlantic coast was likely disrupted due to ice and permafrost. However, post-glacial recolonization pathways and the availability of suitable habitats have allowed sea beet populations to recolonize Europe descending from Atlantic-Mediterranean refugia.^5^ The distribution pattern is closely linked to its modes of pollen and seed dispersal facilitated by marine processes, particularly high tides and oceanic currents.^6^ Typically, the seeds of *B. v*. *maritima* are scattered near the parent plant primarily through the force of gravity.^7^ While seeds also have the potential for long-distance dispersal through hydrochory and their floating abilities, pollen dissemination is wind-mediated and spatially confined.^6^

The genetic resources for both wild and cultivated beets are expanding at a fast pace. At this time, multiple assembled sugar beet genomes have been generated^8^^,^^9^^,^^10^ as well as reference genomes for sea beet,^11^^,^^12^ and *B. patula*.^12^ Recent studies presented re-sequenced genomes of over 650 beet accessions^13^^,^^14^ comprising 265 sea beets (*B. v. maritima*), 45 accessions of *B. v. adanensis*, 30 accessions of *B. macrocarpa,* three accessions of *B. patula,* and 313 sugar beet (*B. v. vulgaris*) accessions. It has been found that sea beets can be divided into a Mediterranean and an Atlantic subgroup according to their genetic distances and geographic origin. This notion was also made earlier based on geographically restricted allozymes and morphological differences^15^^,^^16^ and using Diversity Arrays Technology (DArT) markers.^17^ The study of Sandell et al.^13^ showed that sugar beet is genetically closer to Mediterranean subtypes of *B. v. maritima* than to Atlantic ones using whole-genome sequencing data. In phylogenetic analyses, it turned out that a number of accessions were insufficiently classified, and new (sub-)species assignments were suggested.^13^ The geographic distribution of wild beets has previously been displayed together with profiles of genomic admixture.^13^^,^^14^ However, the geographic coordinates of wild beets were not always accurate, and admixture was only interpreted for a subset of accessions. Thirty of the re-sequenced sugar beet accessions represented modern breeding lines provided by three plant breeding companies, and 48 accessions were derived from a non-commercial sugar beet breeding program. While these sugar beets were observed to cluster together in phylogenetic trees, no further analysis was performed to distinguish them based on their genomic variation or to identify variants that may be specific to one of the subgroups. Global comparisons of wild beets and cultivated beets led to the identification of genomic regions that may be involved in domestication.^8^^,^^14^ However, such areas comprised in total 50 Mbp^8^ or 15 Mbp,^14^ respectively, which was too large to identify a manageable number of genes of interest that were primarily targeted by artificial selection.

Studies on a large number of re-sequenced genomes usually employ genome-wide association^18^ but there are further approaches that can be applied to analyze genomic variation and to find the most important variants that distinguish groups of accessions.^19^^,^^20^ For this work, we decided to use "random forests",^21^ which consist of a large number of "decision trees" each using a different randomly selected subset of the genomic variants. The tree structure arises from the sequential assessment of the variants, asking to what extent they reflect the correct classification of accessions. The result of each tree is a set of variants that performs best in classifying the accessions correctly, and the combination of all trees builds the "forest" that serves as a prediction model based on the most frequently detected informative variant combinations. The reasoning behind using decision-tree based models when working with whole genome sequencing data is their robustness for tasks where the feature-to-sample ratio is heavily skewed toward the features,^22^ in our case, millions of genetic variants (features) and only a few hundred samples. While machine learning algorithms are widely used in most scientific fields that rely on statistical modeling to extract information from complex datasets, they are still underutilized in genomic prediction. We could previously show that random forests outperformed linear mixed models as used in genome-wide association studies (GWAS) when it comes to the prediction of complex phenotypes based on whole genome sequencing data.^20^ In times of ever growing genetic datasets, the efficient usage of artificial intelligence is expected to play an important role in the future of genomics.^19^

Here, we revised the geographic information of *B. v. maritima* accessions by evaluating individual database entries in order to establish a set of geographically localized wild beet accessions that is as comprehensive and accurate as possible. We related genetic to geographic distances by introducing a method for assessing coast-line distances rather than linear distances. We revisit previous re-assignments of beet accessions to (sub-)species using admixture information and discuss further insights from beet admixture. Using random forests, we search for genomic variants that are capable of distinguishing beets of different provenance, including wild and cultivated beets as well as specific subgroups thereof. Lastly, we extract the most important variants that contribute to the distinctions and assess the functionality of the associated genes in the light of beet breeding and domestication.

## Results

### Geographic distribution and genetic distance of wild beets

We inspected the available information regarding the sampling locations of a total of 237 sea beet accessions in public seed banks and manually adjusted geographic coordinates in cases of discrepancies in descriptions and locations (Table S1). The updated geographic coordinates were used to analyze geographic and genetic distances by comparing ancestry proportions^14^ of pairs of beets with linear distance and coastline distance.

**Figure 1:**
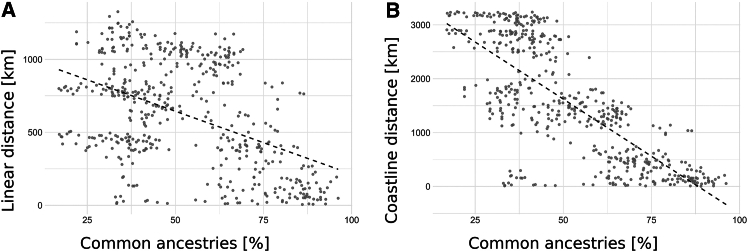
Correlation of genetic and geographic distances Pairwise genetic distances were plotted against linear distances (A) or coastline distances (B) for B. v. maritima accessions from France, Spain, and Portugal. The coastline was defined with a width of 0.8°. French accessions from latitudes above 46°, accessions from Madeira and Corsica, and the accession PI 540574 were excluded. See also Table S2 and Figure S1.

To highlight seed dispersal modes, we focused on accessions from France, Spain, and Portugal below a latitude of 46° in order to analyze datapoints from North-West France following the shoreline to the Strait of Gibraltar and further along Spain to the South of France. The correlation coefficient between the pairwise genetic distance and linear geographic distance was much lower (−0.46) than between pairwise genetic distance and the coastline distance (−0.77) when defining a coast width of 0.8° (with 1° corresponding to about 111 km) (Figure 1; Table S2).

Additionally, genetic and geographic distances were determined relative to the accession PI 540563 from Southern France, chosen as a reference point in the data subset due to its southernmost and easternmost position in France along the shoreline. The correlation between genetic distance and coastline distance was found to be much stronger (−0.86 to −0.88, depending on coastline definition) than the correlation using linear distances (−0.55) (Table S2). The genetic distance to accession PI 540563 increased with increasing geographic coastline distance (Figure S1), and accessions from North-Western France showed the least amount of shared ancestries with the South-Eastern PI 540563 confirming that seeds disperse along the coasts by currents or tides rather than crossing the French inland and indicating that spatial analyses may underestimate the travel distance of seeds to establish subpopulations.

### Re-classified samples and duplicates

A number of 46 beet accessions were suggested to be taxonomically re-classified based on phylogenetic trees in a previous study.^13^ Due to the position of these accessions in the tree, e.g., within a subtree or closer to the root of the tree, there remained uncertainties about the accurate (sub-)species assignment in some cases as also stated by Felkel et al.^14^ based on PCA and admixture analysis. Here, we revisited the genetic admixture profiles and generated pie plots for 667 accessions as an overview and lookup figure (Figure S2). Typical *B. v. maritima* accessions had predominant fractions of "ancestry 12" and "ancestry 4" (Mediterranean accessions) as well as "ancestries" 13, 7, and 5 (Atlantic accessions), whereas *B. v. adanensis* accessions were clearly dominated by "ancestry 1", *B. macrocarpa* by "ancestry 2", and *B. patula* by "ancestry 14" (ancestry numbering as in Figure S3). The remaining eight ancestries were dominant in the cultivated beet accessions. We extracted the accessions that were suggested to be reclassified (see Table 2 in Sandell et al.^13^) and presented them in a separate figure to support the decision on whether reclassification was justified (Figure S3). In several cases there were mixed ancestries so that a clear species assignment remains difficult: BETA 591, BETA 2177, PI 546441, PI 604518, and PI 604545 seem to be hybrids between *B. v. maritima* and *B. v. adanensis* according to admixture plots and BETA 6, BETA 7, BETA 1101, BETA 1344, BETA 1550, PI 121838, PI 504178, PI 535835, and BETA 1228 seem to be hybrids between *B. v. maritima* and *B. v. vulgaris*. In a previous study, BETA 1228 was found to show an atypical mitochondrial genome for a cultivated beet,^23^ which is consistent with our findings. Re-classifications of further accessions as suggested by Sandell et al.^13^ were confirmed by admixture profiles.

**Figure 2:**
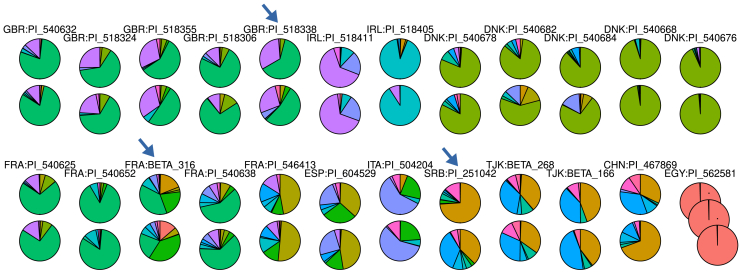
Admixture profiles of 24 accessions that were stored and sequenced separately (23 as duplicates, one as triplicate) The admixture proportions of GBR:PI_518338, SRB:PI_251042, and FRA:BETA_316 (arrows), and of the five duplicated accessions from Denmark (first row, right side) indicated genetic differences as the reason why they were not clustered together in pairs in a previous phylogenetic analysis. Each of the corresponding two (or three in case of EGY:PI_562581) admixture plots is placed underneath each other with their joint identifier on top. See also Figures S2 and S3.

The seeds of 24 beet accessions had been obtained from seed banks two times (in one case, three times) under the same identifier. These accessions, considered as duplicates, were used to test the accuracy of MASH distances^24^ for phylogenetic tree construction^13^ and were not kept for further phylogenetic analyses in Sandell et al. nor in Felkel et al.^14^ The tree containing only such duplicates placed most of them as pairs together and grouped them by country of origin.^13^ However, some accessions with the same identifier were placed apart from each other, i.e., PI 518338 (GBR), PI 251042 (SRB), BETA 316 (FRA), and all five duplicates from Denmark. Inspection of the admixture profiles confirmed that there seem to be larger genetic differences between some of them, so that they actually cannot be considered as true duplicates (Figure 2). Almost all the accessions that were clustered as pairs in the tree showed a highly similar admixture profile, in contrast to the ones that were not placed together and consistently showed divergent admixture proportions. Cases of successful clustering as pairs despite a differing admixture profile, e.g., PI 467869 (CHI) and PI 504204 (ITA), were likely attributed to the geographic separation rather than to a low genetic distance among such pairs. Thus, we consider the accuracy of MASH distances^24^ to be even higher than previously estimated, since the genomic differences between the closely related accessions were correctly reflected in the resulting tree.

### Clustering of admixture profiles

Following the order of accessions in the previously published phylogenetic tree,^14^ main groups of admixture profiles became also apparent in the collection of admixture pie charts (Figure S2): *B. macrocarpa* and *B. patula* were next to each other (first two rows in the figure), *B. v. maritima* was grouped by region indicated by different ancestries, and *B. v. adanensis* appeared with its own ancestry including hybrid forms that show ancestries of both subspecies. However, for cultivated beets, the clustering in the tree did not match the grouping as suggested by admixture very well (lower half of pie charts in Figure S2), except for two groups in the last two rows of the figure ("ancestry 8" and "ancestry 16").

**Figure 3:**
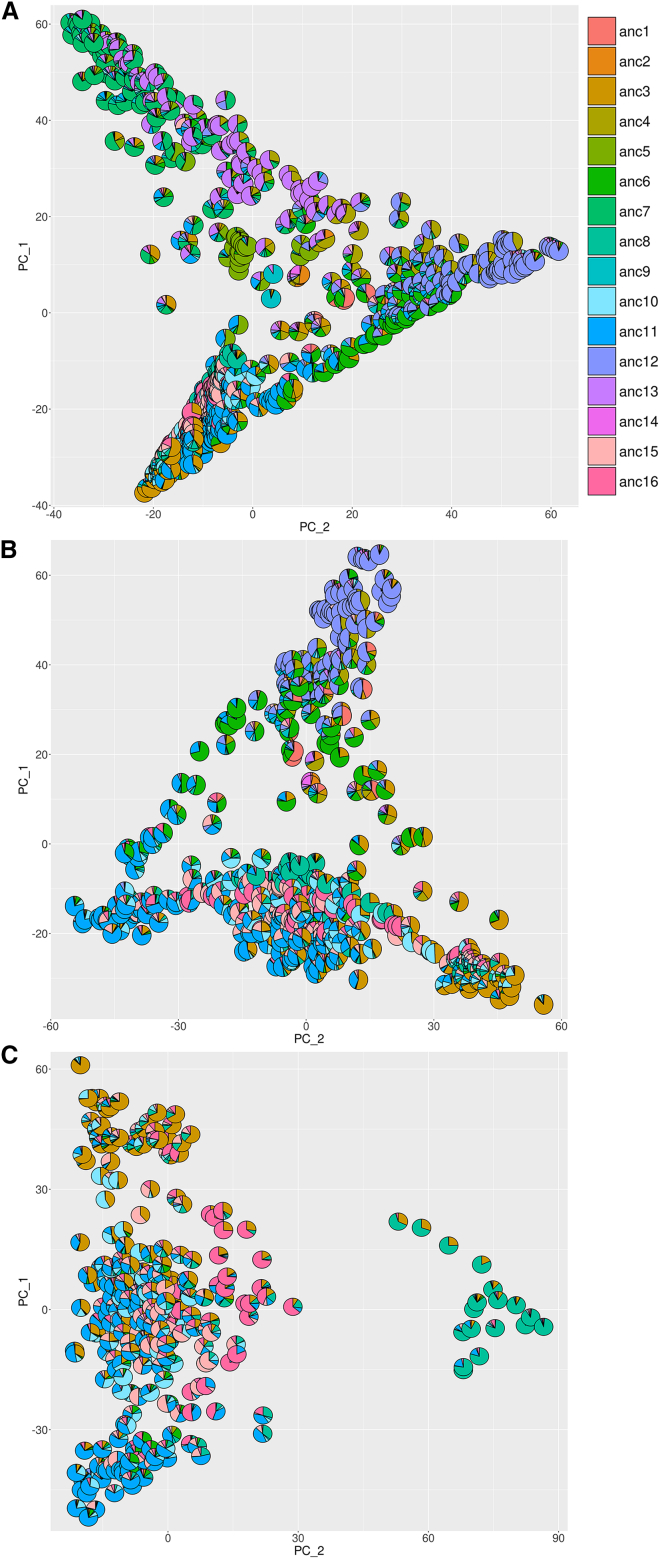
Principal component analysis (PCA) based on admixture proportions (A) All sea beet and sugar beet accessions in the dataset. Atlantic sea beets cluster in the top left area, Mediterranean sea beets cluster in the right area, and sugar beets cluster in the bottom left area. (B) Mediterranean sea beet accessions (top) and sugar beet accessions (bottom) with transition from sea beets to sugar beets via "ancestry 6" (green). (C) Sugar beet accessions only. A distinct cluster dominated by "ancestry 8" appears. See also Figure S4.

We used the admixture information to perform principal component analyses (PCA) for all *B. v. maritima* and *B. v. vulgaris* accessions together, as well as for cultivated accessions separately, to examine their clustering in more detail and independently of a tree structure that only allows bifurcations (Figures 3 and S4).

The PCA of all sea beets and sugar beets highlighted the closer relationship of sugar beets to Mediterranean sea beets rather than to Atlantic sea beets (Figure 3A) in concordance with previous phylogenetic trees. Specifically, the clustering suggested that Mediterranean sea beets (right area in Figure 3A) were ancestors of Atlantic sea beets on the one hand (top left area in Figure 3A) and sugar beets on the other hand (bottom left area in Figure 3A). However, when removing Atlantic sea beets, the clustering indicated two distinct relationships between Mediterranean sea beets and cultivated beets, one via "ancestry 3" and another one via "ancestry 11", with the joint "ancestry 6" representing the transition between wild and cultivated beets (Figures 3B and S4A). After removal of all wild beets, the sugar beets from GBR with dominating "ancestry 8" were clearly separated (Figure 3C). Without these, another group of sugar beets from GBR (dominant "ancestry 16") formed a group fairly distinct from the remaining accessions (Figure S4B). Lastly, when removing these GBR accessions the remaining sugar beet accessions showed one cluster dominated by "ancestry 3", one dominated by "ancestry 11", a third cluster composed of both of these two ancestries, and a fourth one containing mainly "ancestry 10" and "ancestry 15" (Figure S4C). The latter ancestry was dominant in nearly all modern sugar beet accessions obtained from three beet breeding companies. Selecting for all accessions that show "ancestry 15" of at least 27% captured all accessions from Strube (27.8%–49.4% "ancestry 15"), nine out of ten accessions from KWS (29.4%–57.0%, not selected: accession "Laetitia"), and all ten accessions from Syngenta (30.1%–47.6%). However, there were a few additional accessions in our dataset that matched this criterion, too, but were not explicitly derived from the companies: BETA 993 (DEU), PI 518311 (GBR), and BETA 1262 (TUR). By extending the selection to more than one ancestry proportion, it may be possible to find more sophisticated criteria for matching, and thus predicting, certain groups of accessions based on their genomic admixture derived from variant calls. While this notion that genomic differences can lead to a classification of subgroups of beet accessions was promising, we did not further optimize such selection criteria based on admixture profiles since admixture information does not reveal the genomic regions involved. It would be of utmost interest to trace back the genomic variation to particular genes that manifest the difference between groups of accessions. Therefore, we applied machine learning methods directly on the variant calls to perform such tasks.

### Domestication patterns in beets

**Figure 4:**
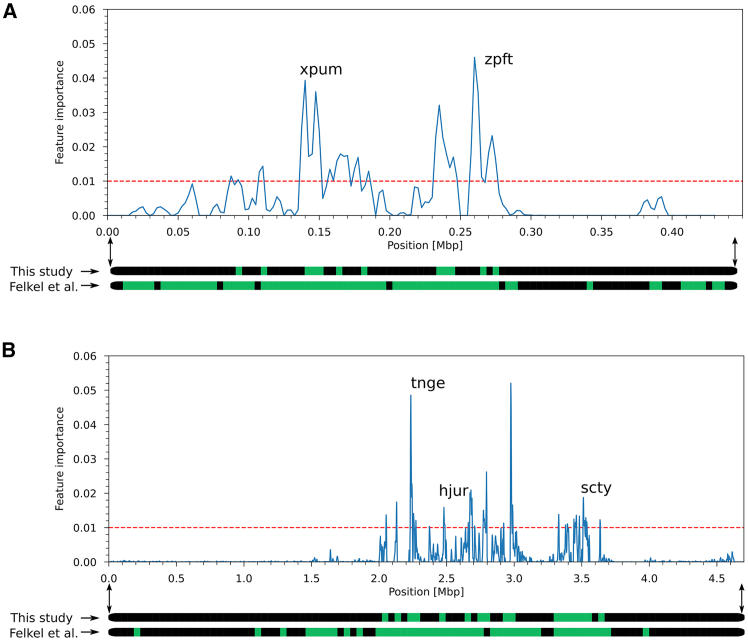
Feature importances of variants distinguishing sea beet and sugar beet accessions in two example scaffolds Example scaffolds of RefBeet-1.2 showing accumulated feature importances of variants (in 5 kbp windows) that distinguish sugar beets from sea beets in chromosome 1 (A) and chromosome 6 (B). Genes related to sugar metabolism in Bvchr5_un.sca010 (re-assigned to chromosome 1) and Bvchr6.sca027 are indicated. Regions assumed to be under selection (at peaks exceeding the dashed line) are displayed in green at the bottom along the scaffolds in comparison to the study by Felkel et al.^14^ where such regions were much larger.

To identify genomic variation distinguishing groups of accessions, we applied machine learning methods to the variant calls. Our first target was to construct predictive models capable of discerning wild beets and cultivated beets. We focused on sea beets as direct ancestors of cultivated beets and within sea beets on the Mediterranean subtype (78 accessions), i.e., the subgroup of wild beets that were determined as genetically closest to sugar beet.^13^ We utilized the ensemble learning method "random forests"^21^ to train prediction models based on genomic variants. We converted the variant call format (VCF) output file as published by Felkel et al.^14^ into a 0/1/2 matrix so that each variant (single nucleotide polymorphism or small insertion/deletion) was classified as either homozygous reference (0), heterozygous (1), or homozygous alternative (2). We calculated 42 distinct random forest models, each trained and tested on different randomly selected subsets of the data. For each model, 75% of the input samples were used for training, and the remaining 25% were used for testing. Each random forest was composed of 5000 decision trees (a number determined through hyperparameter optimization). The number of 42 models was sufficiently large to minimize the likelihood of random effects associated with specific data splits. It guaranteed that each plant was present at least ten times in the testing set. A higher number of models did not lead to a notable impact on the downstream results. For each decision tree, an individual random selection of 5% of the total number of variants was used to minimize the effects of linked variants. Using this approach, we could distinguish wild and cultivated beets with a mean prediction accuracy of 98.4% (for individual model accuracies see Table S3, for misclassified samples see Table 1). The variants that contributed most to the successful classification were kept (i.e., variants of high "feature importance"), ranked by the increase in accuracy directly linked to them. The top list of best variants of the models according to their feature importance value and including at least one best variant of each model comprised 186 different variant positions in the reference genome RefBeet-1.2,^8^ 69 of which were located in scaffold Bvchr1_un.sca014, 31 on scaffold Bvchr2.sca001, 28 on Bvchr2_un.sca008, 15 on Bvchr6.sca027, and 14 on Bvchr5_un.sca010 (Table S4). Further scaffolds had five or fewer variants. Assuming that artificial selection during the formation of sugar beet, i.e., the process of domestication, is reflected in genetic intervals rather than individual positions, we summed up the feature importances of variants within 5 kbp windows along the reference genome sequence. The resulting peaks were considered as indicative of genetic regions associated with domestication and/or diversification patterns (Figure 4).

**Table 1:** Misclassified accessions in random forest models distinguishing sea beets from sugar beets

Misclassified samples in models for domestication patterns (sea beets vs. sugar beets)		
15	PI 604513	sea beet
13	PI 116809	sugar beet
10	BETA 1228	sugar beet
10	PI 604512	sea beet
9	PI 604523	sea beet
6	PI 546538	sugar beet

The first column specifies in how many of 42 models the accession given in the second column was misclassified, the third column indicates if the accession was a sea beet or a sugar beet.

The study by Felkel et al.^14^ used the bolting locus (*B*-locus) as starting point to define the properties of a genomic region involved in beet domestication based on the knowledge that all cultivated beets are biennial plants.^25^ We checked the accumulated feature importances as determined by random forests in the region of the *B*-locus and only found a relatively low signal. Closer inspection revealed that no variant reliably separated cultivated beets from wild beets in the region of the *B*-locus, most likely because biennial sea beets were also represented in the set of accessions. To substantiate this notion, we built a random forest model based exclusively on variants within the B-locus, resulting in a prediction accuracy of 85%, far below our initial models. Thus, the *B*-locus does not represent a reliable target for the separation of sea beets and sugar beets and was not further taken into account in this study to select a threshold for domestication signals.

We selected genomic regions that displayed a 2.5 times higher signal than the average cumulative feature importance value of variants per window to distinguish signal from noise. Genes in such windows were considered to have a high certainty of being domestication-related genes or involved in sugar beet diversification compared to other cultivated beets.

In cases where two or more consecutive windows exhibited signals above the cutoff, we combined these windows into a single region. In total, we identified 173 regions (475 windows) corresponding to a total genomic length of 1.67 Mbp (range 5–27.5 kbp per region) encompassing 174 genes presumably related to domestication or diversification (Table S5).

We compared the locations of these regions with the signature of artificial selection previously reported by Felkel et al.^14^ Nearly all of the regions that were identified using the machine-learning approach were situated within these previously identified regions (Figure 4), but only at a size of 11% of the previously identified 15 Mbp. Thus, the machine learning approach could substantially narrow down the candidate regions and number of genes due to the emergence of distinct peaks directly at the decisive variations between sugar beets and sea beets.

We performed functional annotation for all candidate genes based on homology searches. We found an array of functions that would indeed be expected for genes associated with sugar beet domestication or diversification, i.e., functions related to sugar metabolism, environmental adaptation, and pest defense, as well as regulating growth and organ development (Table S5).

Specifically, we identified six genes directly implicated in sugar metabolism. Among them, one gene, Bv1_015950_zugk, was located on Bvchr1.sca008, two genes, namely Bv5_124820_xpum and Bv5_124860_zpft, were situated on Bvchr5_un.sca10 (comparison to the sugar beet assembly of genotype EL10^9^ showed that this sequence also belongs to chromosome 1), and three genes, Bv6_150380_hjur, Bv6_151020_scty, and Bv6_150160_tnge, were located on Bvchr6.sca027 (Figure 4). According to their assigned functions, Bv1_015950_zugk encodes an ERD6-like sugar transporter, which was linked to the response to drought stress,^26^ Bv5_124820_xpum (LOC104908065) is a homolog of chloroplastic hexokinase-2 and may be involved in regulating sugar accumulation in sugar beet. The third gene, sucrose transport protein SUC4 (Bv5_124860_zpft, LOC104908070) encodes a phosphoglucomutase (PGM), which is known to be important for starch synthesis in dicots.^27^^,^^28^ Earlier studies have shown that transcript levels of this gene are significantly increased in taproots^29^ and that plastidic PGM is 23 times more abundant in parsnips than in sugar beets.^28^ The transcription of this gene is increased under cold condition, and it was hypothesized that SUC4 in taproots is the driving force in the accumulation of sugar and its delivery to shoots.^29^ These results indicate that SUC4 is one of the major domestication genes in sugar beet, leading to enhanced sugar accumulation in the roots. Bv6_150380_hjur (LOC104897526) was annotated as glucose-6-phosphate dehydrogenase encoding an adenylate kinase. In humans, adenylate kinases regulate blood sugar levels by controlling K-ATP channels, thereby promoting insulin secretion.^30^ The assigned function of Bv6_151020_scty (LOC104897586) was mannosyl-oligosaccharide glucosidase GCS1. The gene Bv6_150160_tnge is a homolog of SWEET15^31^ and encodes a bidirectional sugar transporter that has been shown to increase hexose sugar accumulation in common grapevine.^32^

Apart from genes related to sugar metabolism there were several other genes with notable functions located in genomic regions that distinguished sugar beets from wild beets according to feature importances of the variants: on the one hand Bv6_150600_fzwz (LOC104897547) annotated as histidine kinase 3, a signal transducer responding to water scarcity, salt-induced stress, bacterial infection, and low temperature and presumably regulating flower development and seed germination; on the other hand genes with functions related to nematode resistance (Bv5_124870_ydpo), bacterial resistance (Bv6_130550_saor, Bv1_003350_wzzj, Bv6_150600_fzwz, Bv7_175040_odrg), flower and root development (Bv1_022070_hgze, Bv7_175040_odrg), anther and stomium development (Bv6_130720_rkjk), and root growth (Bv2_046570_sjfj, Bv1_022070_hgze).

We compared our results to the approach of classical genome-wide association studies (GWASs) implemented as linear mixed models (LMMs) in GEMMA.^33^ After applying a stringent multiple testing correction, we identified significant variants in 3,536 genes, which is 20 times more than those identified by the random forest approach. Of these, 24 genes were common to both methodologies. Notably, this set of shared genes included three of the six identified sugar-related genes: SWEET15, SUC4, and GSC1.

### Selection patterns in modern sugar beet breeding lines

Among the 292 sequenced sugar beet accessions, 48 accessions were pre-breeding material from the USDA beet breeding program at East Lansing, and 30 accessions were modern breeding lines obtained from the companies KWS SAAT SE, Strube Research, and Syngenta. The remaining 214 sugar beet accessions were from obtained from public seed repositories at the USDA and IPK. We built predictive models using random forests to distinguish modern breeding lines provided by companies from the remaining sugar beet accessions (Table S3). The mean accuracy of the models was 91.7%, indicating that modern sugar beet breeding material exhibits a distinct profile that enables its differentiation from other sugar beets. Closer inspection revealed that the accessions misclassified by the models were PI 518311 and accessions from the East Lansing pre-breeding program. PI 518311 exhibited a fairly similar admixture profile to that of accession "Beretta" from KWS (Figure S2). When recalculating our models without PI 518311 and without accessions derived from East Lansing, the mean prediction accuracy of the models increased to 98.5%. Accessions still misclassified by some models included two from India and the US, respectively, and four from KWS (Table 2). In contrast, all Strube and Syngenta accessions, as well as all remaining public accessions, were classified correctly in all models.

**Table 2:** Misclassified accessions in random forest models distinguishing sugar beets available in public seed repositories from sugar beet breeding lines obtained from seed companies (KWS, Strube, Syngenta)

Misclassified samples in models distinguishing public from company sugar beets		
11	PI 116809	public
9	PI 605413	public
8	Malvina	KWS
7	Vivianna	KWS
2	Evelina	KWS
2	Pauletta	KWS

The first column specifies in how many of 42 models the accession given in the second column was misclassified, the third column indicates where the accession originated from.

The feature importances of the variants detected as informative by the models were analyzed in 5 kbp windows (see above, see STAR Methods), revealing the by far strongest signal on scaffold 5 of chromosome 3 in RefBeet-1.2 (Figure S5). Here, a large number of variants accumulated within a very short stretch of the reference genome, pointing to a specific region that distinguishes modern breeding material from other sugar beets. The gene Bv3_057020_mahi (Bvchr3.sca005:2228571.2248735), functionally annotated as encoding a mediator of RNA polymerase II transcription subunit 25 (LOC104888788), was identified to be located directly at the peak of the signal, and a total of 49 variants with feature importances greater than zero were detected within the gene (Table S6). According to gene ontology (GO) annotation,^34^ Bv3_057020_mahi is linked with the GO term GO:0050832 described as “response to the presence of fungus that acts to protect the cell or organism,” suggesting a role of this gene during fungal infection. The variants located within the gene showed a homozygous reference status for public accessions in this region. In contrast, the modern breeding lines were either heterozygous or homozygous for the alternative allele at these positions.

To identify possible wild donors of this trait characteristic for modern breeding lines, we searched the variant pattern of Bv3_057020_mahi in the genome of sea beet (*B. v. maritima*)*.* In the variant call data of 237 sea beets,^14^ we discovered eight accessions that showed the exact same variant pattern as the modern sugar beet accessions (Table 4). All of these wild beet accessions originated from Italy, more specifically, from Southern Italy (Calabria, Basilicata) or from Sardinia.

**Table 4:** Sea beet accessions sharing a variant pattern within the fungal resistance candidate gene Bv3_057020_mahi with modern sugar beet breeding material

Accession	Country	Region	*Erysiphe* Resistance
BETA 1002	Italy	NA	NA
PI 504198	Italy	Calabria	NA
PI 504210	Italy	Calabria	NA
PI 504212	Italy	Calabria	4
PI 504216	Italy	Basilicata	0
PI 504220	Italy	Sardinia	1
PI 504231	Italy	Sardinia	0
PI 504249	Italy	Sardinia	0

*Erysiphe* resistance is annotated on a scale from 0 (immune) to 9 (highly susceptible) following the publicly available USDA GRIN database. NA: not available. See also Table S6.

We surveyed the publicly available disease resistance annotations of the USDA GRIN database for these wild beets and found that five of eight accessions had previously been screened for *Erysiphe* resistance, and four of them had either been annotated as immune or highly resistant to *Erysiphe* infection. Thus, it seems that the gene showing accumulated differences between wild beets and sugar beets plays a role in *Erysiphe* resistance and had been selected explicitly for in all modern sugar beet lines analyzed in our study.

Among the top 25,000 variants identified by the models throughout the genome (sorted by feature importance) about 15,000 variants were located on Bvchr3.sca005. The next most frequent number of highly important variants was found on Bvchr3.sca006 (about 3700 variants among the top 25,000), pointing to a neighboring genomic region on chromosome 3, which may be a result of little recombination in the targeted region due to linkage drag. Further scaffolds had much lower numbers of variants (e.g., the next highest number was on Bvchr5.sca023 with 878 variants). At these locations, there may be further genes characteristic of the modern breeding lines, but none of the variants elsewhere were reported due to the exceptionally strong signal at the Bv3_057020_mahi locus on chromosome 3 (see discussion).

### Difference in breeding efforts

Since the set of sugar beet accessions obtained from breeding companies were clearly distinguishable from other sugar beets we went one step further and trained random forests to predict which company an accession had been obtained from. This means we tried to identify regions that were unique in accessions of a single company, i.e., presumably regions where breeding efforts had focused on. We calculated random forests as described above for each pairwise combination of companies, resulting in models with a mean prediction accuracy of 94%. The distinction between accessions from KWS and Strube was most successful with a mean prediction accuracy of 97.1%, followed by the split between Strube and Syngenta with a mean prediction accuracy of 96.5%. Our models were least successful in differentiating KWS from Syngenta accessions with a mean prediction accuracy of 88.1%. Since the sample size of 10 accessions per company is small, the performance of a model is even more dependent on the choice of training and test sets than in models with more samples. Some accessions seem to be less specific to the companies so that a training set containing such accessions will result in a model that performs worse, and we observed a prediction accuracy as low as 60% in the worst models (Table S3). Nevertheless, the high mean accuracy achieved indicated that it was possible to separate the groups based on company-specific genomic regions clearly. The three sugar beet accessions that were misclassified most often were Cardamone (Syngenta), Laetitia (KWS), and Pauletta (KWS) (Table 3). In these cases Pauletta was classified as an accession originating from Syngenta and Cardamone was classified as a Strube accession, possibly indicating an exchange of breeding material. K-mer based phylogenies^13^ had also placed Cardamone inside the Strube subtree and Pauletta inside the Syngenta subtree. Laetitia from KWS (in some models misclassified as accession from Syngenta) showed a rather distinct admixture profile compared to all other company accessions (Figure S2) and was placed apart from KWS accessions in phylogenetic trees.^13^^,^^14^

**Table 3:** Misclassified accessions in random forest models distinguishing sugar beet breeding lines by the company they were obtained from (KWS, Strube, Syngenta)

**Misclassified samples in models for KWS vs. Strube**		

3	Laetitia	KWS
3	Premiere	Strube

**Misclassified samples in models for KWS vs. Syngenta**		

7	Pauletta	KWS
5	Evelina	KWS
3	Contenta	Syngenta
3	Laetitia	KWS
2	Cardamone	Syngenta
2	Gerty	KWS
2	Rasta	Syngenta
1	Malvina	KWS

**Misclassified samples in models for Strube vs. Syngenta**		

6	Cardamone	Syngenta
1	Premiere	Strube

The first column specifies in how many of 42 models the accession given in the second column was misclassified, the third column indicates which company the accession originated from.

For each company pairing (KWS-STR, KWS-SYN, SYN-STR), we analyzed the feature importances of the variants that were decisive in splitting accessions of one company from accessions of the two other companies. We focused on genetic windows of 5 kbp that exhibited an increased number of variants with high feature importance (see STAR Methods). The identified windows covered a total of 259 genes (84 KWS, 121 Strube, and 54 Syngenta) that demonstrated substantial differences among the breeding companies (Table S7). This methodology allows for a comprehensive detection of specific regions within the sugar beet genome that serve as targets for breeding efforts. For instance, the only gene that differed among all three breeders was the gene Bv3_062780_rmep. The variants that distinguished the alleles had a high mutational effect: of seven mutations in total, two and five variants affected splice donor sites or splice acceptor sites, respectively, at least resulting in the abnormal splicing of the transcript, or, more likely, gene inactivation. Gene Bv3_062780_rmep is homologous to the MADS-box transcription factor APETALA1. In *A. thaliana,* this is a well studied gene that controls how flowers and petals are formed.^35^ In sugar beet, this gene has been found to be differentially expressed during vernalisation^36^ which may be the reason why this gene was a target of artificial selection.

The functions of genes carrying variation that was exclusive to one of the three breeding companies encompass a diverse range of processes, including carbohydrate metabolism, heat and cold responsiveness, bract formation, regulation of the timing of transition from vegetative to reproductive phases, and lateral root growth (Table S7).

## Discussion

In this work, we analyzed re-sequencing data and genetic variation in beets of the genus *Beta* to investigate genetic differences among wild beets, between wild and cultivated beets, and among cultivated beets. We used innovative data analysis methods and inspected the data from different angles that were not considered in previous studies. Our work showcases how analyzing publicly available data in more depth and with different methods can result in new insights that go beyond previous findings. Such approaches are of general interest in times when sequence repositories keep growing at an unprecedented pace. While data generation has now been streamlined and can be efficiently done for large numbers of samples^37^ novel application of data analysis methods is crucial to extract specific biologically meaningful information.

To enhance the value of the available sequencing data for subsequent interpretation, we first revised the collection site information and geo-coordinates of the wild beet accessions used in this study. Using the improved geographic information, we studied the relationship between genetic distance and geographic distances of *B. v. maritima* to get a better understanding of the propagation of wild beets. We correlated genetic distances with linear geographical distances and seven scenarios of coastline distances, modeling the extent at which seeds can disperse into the open sea. Our results revealed a superiority in correlation between genetic distance and coastline distances with broader coastal width over linear distances indicating that coastlines and open sea act as conduits for the dispersal of wild beet seeds. In contrast, the weaker correlation with linear distance suggests that land-based dispersal mechanisms, such as the distribution of pollen by air currents or the spread of seeds mediated by animals, are of minor importance. Thus, our data suggest that the distribution of genetic diversity does not follow geographic distributions over land, but rather along the coastline, contrasting findings by Andrello et al.^17^

The highest correlation coefficient for pairwise comparison of genetic and coastline distance was observed in the scenario with a coastal width of 1.2° for the full dataset and 0.8° for the subset, indicating that seeds can be dispersed far into the sea. In contrast, scenarios that measure coastlines closer to the shore tend to overestimate distances. Fievet et al.^6^ also found that coastline distance better represents isolation-by-distance patterns of a microsatellite-based dataset than the topography of the coasts.

While the Strait of Gibraltar has been previously identified as an efficient barrier to species propagation,^38^ there are also indicators of genetic exchange occurring beyond the Strait of Gibraltar for *B. v. maritima*.^5^ However, only comprehensive sampling can conclusively determine the extent of genetic exchange, whether it is continuous or sporadic.

By inspecting genetic admixture in the 46 samples that were suggested to be re-assigned to another (sub-)species (see Table 2 in Sandell et al.^13^), we could confirm the new assignment of 32 accessions but also showed for which 14 accessions species assignments remained unclear (Figures S2 and S3). In case of hybrids between *B. v. maritima* and *B. v. adanensis* one cannot tell whether or not these are artifacts due to propagation *ex situ*. The confirmation that natural hybrids exist would require the sequence information of single plants from the accessions' place of origin. Hybridization between *B. v. maritima* and *B. v. vulgaris* is known to occur frequently.^2^

Admixture profiles also revealed why accessions that were considered as "duplicates" did not cluster as pairs in previous phylogenetic analysis^13^ based on MASH distances.^24^ We combined PCA and admixture analysis to resolve the relationship between groups of cultivated and wild beets and also showed that groups of accessions can be successfully recognized based on their admixture proportions as exemplified by a characteristic "ancestry" for modern sugar beet breeding lines. This approach may also be used to propose the collection area of wild beets of unclear origin. The classification of groups of accessions based on admixture refers to the genetic basis of their differences, which we directly addressed in subsequent analyses using machine learning.

We applied random forests on the extensive whole-genome variant dataset. We successfully identified specific genomic locations that differentiate 1) wild beets and cultivated beets, 2) publicly available cultivated sugar beet accessions and modern breeding lines, and 3) between three groups of breeding lines obtained from three seed companies.

Several genes that distinguished sugar beets from sea beets were directly linked to sugar metabolism. One of these genes, Bv5_124860_zpft, encodes the sucrose transporter SUC4 that was described to contribute to sugar accumulation in beetroots.^29^ Additionally, it has been shown that SUC4 plays an important role in root growth during high sucrose conditions in *Arabidopsis*.^39^ These properties suggest that SUC4 is an important gene in sugar beet domestication and diversification, not only regulating sugar storage in the roots but also allowing for strong root growth despite unfavourably high sucrose conditions. Our findings demonstrate that the random forest methodology produces a more targeted outcome compared to linear mixed models (LMMs). Specifically, the traditional genome-wide association study (GWAS) approach identified over 20 times more significant genes than the random forest method; however, GWAS failed to detect three genes directly associated with sugar metabolism in cultivated sugar beets that were identified by the random forest approach.

Our machine learning models exhibited a mean predictive accuracy of 98.5% in distinguishing elite breeding lines from sugar beets deposited in public seed banks and revealed a strong signal on sugar beet chromosome 3, namely at the location of a putative *Erysiphe* resistance gene, and this was the only candidate gene reported due to its strong signal. According to shared variation patterns, this gene was likely introduced into modern sugar beets from Italian sea beets originating from around Sardinia. The identified gene was Bv3_057020_mahi, encoding the mediator of RNA polymerase II transcription subunit 25 (MED25) according to its functional annotation. In *Arabidopsis,* MED25 was demonstrated to modulate jasmonate-mediated resistance against *Botryris cinerea*, a necrotrophic fungus commonly affecting grapevine.^40^^,^^41^ Additionally, it was hypothesized that MED25 is indispensable for the jasmonate-mediated defense mechanism against necrotrophic fungal pathogens such as *Erysiphe* that infect leaves in various plant species.^42^ From these findings we conclude that the strong signal identified at the MED25 locus is most likely related to *Erysiphe* resistance in sugar beet.

We also compiled a comprehensive list of genes that highlight the breeding focus employed by specific companies. The dataset used in this study was quite small, consisting of only ten sugar beet accessions per company. It has been shown in the past that random forests are well suited to handle small datasets.^43^ In contrast, GWAS is known to require a large sample size to achieve sufficient statistical power.^44^ The efficacy of decision tree-based models is largely determined by factors such as sample size, the quality of both the predicted phenotype and the predictors, and the homogeneity of the classes. When working with small sample sizes, there is a significant chance that patterns emerging for effective classification may not be directly correlated with the desired phenotype. As a test, we calculated models to distinguish between breeding lines of different companies using random subsamples of seven or five accessions per company, respectively. The results showed a decreased mean prediction accuracy of 88.6% with seven accessions per company and a further decreased accuracy of 82.3% with five accessions, indicating that a decline in accuracy is clearly measurable for these sample sizes, but ten accessions with a high mean accuracy of 94% are still adequate.

Our study provides data-driven approaches that can be used to comprehensively analyze the genetic diversity within both wild and cultivated beet populations. We showed that random forests are not only very robust when a high number of features (variants) is involved but also allow us to reveal complex combinations of variants that determine a trait of interest. Importantly, our methods can also be applied to other crops for which resequencing data are available from a phenotypically diverse set of accessions. This presents new opportunities for research on genotype-phenotype interactions and targeted breeding to enhance crop performance.

### Limitations of the study

While the dataset represents a relatively large collection of sequenced genomes, more comprehensive results could be achieved with an increased number of samples, particularly those reflecting modern breeding material. Another limitation is the scarcity of phenotypic data. With broader and more detailed phenotypic information, our models could be extended to investigate the genetic basis of additional traits, such as resistance to other diseases beyond *Erysiphe*, or even complex polygenic traits such as yield.

A potential shortcoming of our model-based approach in identifying genomic regions under selection is that, due to a very strong signal observed in one particular region, other relevant genes may remain unnoticed. At the gene locus of MED25 on chromosome 3, the machine learning models could very reliably distinguish elite sugar beet lines from other accessions, and this is, technically speaking, due to a large mean impurity decrease for the given gene compared to all others. Hence, for discovering further genes under selection, one would need to remove detected signals iteratively until the separation of the groups based on the remaining variants is no longer possible with high prediction accuracy.

## Resource availability

### Lead contact

Requests for further information and resources should be directed to and will be fulfilled by the lead contact, Juliane Dohm (dohm@boku.ac.at).

### Materials availability

This study did not generate new unique reagents.

### Data and code availability

Analysis scripts were written in Python 3.6 and are available on https://github.com/FLsandell/.

## Acknowledgments

We are grateful to Christian Gottschall for the administration of a Linux compute cluster, J. Mitchell McGrath for discussions, and Thomas Holzweber for support related to functional annotations and for discussions. This work was supported by Österreichische Forschungsförderungsgesellschaft mbH (10.13039/501100004955FFG) grant number 853197 "BeetSelect" and by the Austrian Science Fund (10.13039/501100002428FWF) grant number P 32860-B "CultiBeet." Additional support was provided by the Doctoral School AgriGenomics of BOKU University.

## Author contributions

J.C.D., H.H., and F.L.S. conceived the study, F.L.S., C.R., and J.C.D. analyzed data and drafted the article, J.C.D. and H.H. supervised the work and revised the article. All authors read and approved the final version of the article.

## Declaration of interests

The authors declare no competing interests.

## STAR★Methods

### Key resources table

**Table:** 

REAGENT or RESOURCE	SOURCE	IDENTIFIER
**Deposited data**		

Raw sequencing data	Felkel et al.^14^	NCBI:PRJNA815240
Sugar Beet Reference Genome	Dohm et al.^8^	NCBI:GCF_000511025.2
Geographic coordinates	GBIS/I	https://gbis.ipk-gatersleben.de/gbis2i/
Geographic coordinates	USDA-ARS GRIN	https://www.ars-grin.gov/
BeetSet-2	Minoche et al.^45^	https://bvseq.boku.ac.at/Genome/Download/RefBeet-1.2/
eggNOG v5 database	Huerta-Cepas et al.^46^	http://eggnog5.embl.de/#/app/home

**Software and algorithms**		

R v4.2.1	R Core Team^47^	https://www.r-project.org/
*distGeo*	Hijmans^48^	https://doi.org/10.32614/CRAN.package.geosphere
*QGIS v3.12*	QGIS Development Team^49^	https://www.qgis.org
ggplot2	Wickham^50^	https://ggplot2.tidyverse.org
scikit-learn	Pedregosa et al.^51^	https://scikit-learn.org
GEMMA	Zhou and Stephens^33^	https://github.com/genetics-statistics/GEMMA
vcftools v0.1.16	Danecek et al.^52^	https://github.com/vcftools/vcftools
eggNOG-mapper v2.1.9	Cantalapiedra et al.^53^	https://github.com/eggnogdb/eggnog-mapper
DIAMOND v2.0.15.153	Buchfink et al.^54^	https://github.com/bbuchfink/diamond
BLAST+ v2.15.0	Camacho et al.^55^	https://blast.ncbi.nlm.nih.gov/doc/blast-help/downloadblastdata.html
snpEff v5.1	Cingolani et al.^56^	https://pcingola.github.io/SnpEff/
Custom ML scripts	This study	https://github.com/FLsandell/

### Method details

#### Geographic coordinates

Coordinates of 237 *B. v. maritima* accessions were manually revised based on collection site information in the databases, i.e. in GBIS/I (Genebank Information System of the IPK Gatersleben), in the USDA-ARS Germplasm Resources Information Network (GRIN), and in the International Database for Beta (IDBB) at Julius-Kühn Institut, Quedlinburg, Germany. At the time of writing, IDBB was not maintained anymore but information was downloaded while the site was still accessible and was included in Table S1. Coordinates were kept if the location was in line with the description of the collection site. In many cases the coordinates differed from the description, e.g. pointed towards a location in the sea or in the middle of an urban area although the description said "at the beach". We moved the coordinates to places that we considered a better match with the descriptions, making use of Google Maps (https://www.google.com/maps). In some cases we could only guess (e.g. description "Sicily" with no further detail nor coordinates). If the geographic information was unspecific (e.g. "France") we took admixture plots into account to deduce the presumptive area of origin (e.g. Northern or Southern France). BETA 194 had a collection site described as "Tripoli, Tarabulus, at sea shore" with country code "LBY" but coordinates were pointing to Tripolis, Lebanon. In case of disagreement between sample site description and coordinates, we gave preference to the written descriptions. We commented all accessions and provide updated coordinates along with the ones from the databases (Table S1).

#### Geographic distances

Pairwise geographic distances were calculated for 230 *B. v. maritim*a accessions, for which geographic coordinates were available and which were within the defined geographic extension (-24.750211604, 43.397281899, 27.493343180, 68.131756736 according to EPSG:4326). Accessions from Corse and Madeira, although belonging to France and Portugal, respectively, were excluded due to their geographic locations and distance to the mainland whilst accessions from the islands La Palma and Ibiza were kept in the data set. Accession PI 540574, described as originating from Northern France, was excluded due to the discrepancy between its geographical location and its admixture profile, which indicated a Mediterranean genetic background. First, to simulate long-distance seed dispersal through air or animals, pairwise linear distances were determined in R v4.2.1^47^ using the function *distGeo* of the package *geosphere*^48^*,* which calculates the shortest path on an ellipsoid. Secondly, coastline distances were calculated to represent seed dispersal through water. An open-source shapefile (https://www.naturalearthdata.com/downloads/50m-cultural-vectors/) containing polygons of country outlines was used to create a cost grid which served as basis for this approach. In *QGIS v3.12*^49^ we added a buffer polygon to the seashore line of the country polygons which was defined as *coast*, finally resulting in three types of polygons: *land*, *coast* and *sea*. *Land* was then attributed the value 99999, *coast* the value 1 and *sea* the value 500. The shapefile was rasterized with a 0.05x0.05 resolution of geo-referenced units assigning each resulting grid cell the respective value. Using R (packages *raster*, *rgdal* and *gdistance)*, we then determined the length of the connecting path between each pair of accessions through the grid cells which obtained the minimum overall score. So, the paths followed predominantly coastlines, however, to interconnect islands or continents, the sea was crossed at the narrowest sections to minimise costs. For the modulation of different seed dispersal patterns along the coastline, we proposed seven different widths for the buffer polygon *coast*: 0.05°, 0.2°, 0.4°, 0.6° 0.8°, 1° and 1.2° respectively, with one degree covering about 111 km (Figure S6). Pairwise coastline distances were calculated separately for each of these scenarios. As the width of coast increases, seeds are assumed to travel further out to sea, leading to shorter connection paths.

Correlation plots were generated using R v4.2.3^47^ using the library ggplot2.^50^ The map in Figure S6 was generated with QGIS v3.36.2.^49^

#### Analysis of admixture profiles

We used admixture profiles that had been previously determined^14^ to assess pairwise genetic distances for 230 *B. v. maritim*a accessions. We calculated the overlap in ancestry for each pair and then summed up these values across all 16 ancestries to obtain a final value for genetic relatedness. Admixture pie plots were generated using R v4.2.1^47^ with the libraries ggplot2,^50^ and scatterpie.^57^ PCA plots were generated using the function PCA from the python library scikit-learn.^51^

#### Univariate GWAS

The univariate GWAS was calculated using LMMs as implemented in GEMMA.^33^ For each LMM a centered relatedness matrix was calculated to correct for population structure (−gk 1). The LMMs were calculated using the Wald test as a frequentist (−lmm 1). Each model was then corrected for multiple testing using Bonferroni correction (P < 5.8 × 10^−9^).

#### Genetic variation analysis

Publicly available variant calls^14^ related to RefBeet-1.2^8^ were transformed from a filtered bcf file into a 0 | 1 | 2 matrix using vcftools v0.1.16 with the --012 flag.^52^ This matrix was thereafter used as the input for all genetic variation-related analyses and machine learning based approaches.

For each phenotype (cultivated vs. wild, modern breeding material vs. old seed bank accessions, dinstinct company assignment) random forests were trained using the Python (v3.6) machine learning library scikit–learn v0.24.2.^51^ For each model, the data was split into a training and testing set using train_test_split with a standard train size of 0.25 and stratified sampling. Parameter optimization for the random forests were conducted using a grid search over the parameter space using the scikit–learn function GridSearchCV with five cross-validations (cv = 5) and 1000 iterations. Model accuracies were assessed on the test sets. Feature importances were calculated using the scikit–learn function feature_importances_. The feature importance is based on the accumulation of the impurity decrease within each decision tree.

To identify genomic regions that have a high number of features that separate two groups, a custom Python script was used that sums up the feature importances of all 42 models contained within a sliding window of 5000 bp with an offset of 2500 bp so that each position was covered by two windows. When multiple models detected the same variant, we chose the one with the highest feature importance. The cutoff (0.0104) for determining windows that play a role in domestication was set to 2.5 times the average feature importance (0.0042) for every window that contained at least 10 variants. To identify important windows for differences between accessions from companies we randomised the feature importances for all features detected by our models 10,000 times and selected windows that showed a higher feature importance than 99% of the simulated distributions.

#### Functional annotation of target genes

We functionally annotated the genes of BeetSet-2^45^ that contained important variants distinguishing sugar beets from sea beets using eggNOG-mapper v2.1.9^53^ with DIAMOND v2.0.15.153^54^ and the eggNOG v5 database^46^ and analysed the resulting gene ontology (GO) terms.^34^ For genes distinguishing other groups of accessions we referred to the functional annotation published with RefBeet-1.2^8^ and performed blast searches using BLAST+ v2.15.0^55^ and the NCBI nr database as of May 2024 with default parameters. The functional impact of identified variants was analysed using snpEff v5.1.^56^

#### Computing resources

Programs and commands were run on a Linux computing cluster featuring a CentOS 6.7 operating system with nine computing nodes equipped with between 24 (2.6 GHz) or 56 (4.0 GHz) cores and a maximum of 256 GB RAM.

### Quantification and statistical analysis

For the univariate GWAS conducted using GEMMA, p-values of the identified variants were adjusted for multiple testing using the Bonferroni correction, with a significance threshold set at 0.05. After correction, variants with p-values below 5.8 × 10^-9^ were considered statistically significant.

For the machine learning models, a window was considered relevant to domestication if its feature importance exceeded 2.5 times the average feature importance (0.0042), resulting in a cutoff value of 0.0104. Only windows containing at least 10 variants were included in this analysis. To identify windows associated with differences between accessions from different companies, we performed 10,000 random permutations of the feature importance values. Windows with feature importance values exceeding the 99th percentile of the permuted distribution (p < 0.01) were considered significant.

## Supplemental information

Document S1. Figures S1–S6

Table S1. Manually curated geographic coordinates of 237 sea beet accessions; related to STAR Methods Content of columns 1–7: Accession: accession identifier; Species: taxon assignment including re-assignments as proposed by Sandell et al. and discussed in this study; Source_coord: source of geographical coordinates and whether they were the same if present in more than one database (indicated by "DIFF" or " = "); Country: country of origin of the accession; Lat_Final and Lon_Final: final latitude and longitude after manual curation; Coordinate_info: curation comment. The following columns contain information from IDBB, IPK, USDA (indicated in the column head) and converted coordinates in decimal degrees.

Table S2. Correlation coefficients for pairwise genetic distance vs. linear distance and vs. seven coastline distances for the full dataset of sea beet accessions, a subset from France, Spain, and Portugal, and for the same subset in relation to PI 540563; related to Figures 1 and S1 Coastline distances were calculated using paths defined by varying buffer widths around the coastline, ranging from 0.05° to 1.2° (with 1° corresponding to about 111 km). For each dataset, the strongest negative correlation is highlighted in yellow.

Table S3. Prediction accuracies for 42 computed random forest models to distinguish cultivated from wild accessions, breeding material from seed bank accessions, and company provenance (KWS, STR = Strube, SYN = Syngenta); related to Table 1

Table S4. The top list of best variants of the models distinguishing Mediterranean sea beets from sugar beets according to their feature importance value, and including at least one best variant of each model; related to Figure 4 Scaffold names and positions refer to sugar beet reference assembly RefBeet-1.2.

Table S5. Genomic regions in RefBeet-1.2 and variants (feature importance >0.0004) distinguishing sugar beets and sea beets; related to Figure 4 Genes linked to variant positions are listed including functional annotation.

Table S6. Variant positions in the proposed Erysiphe resistance gene (and 1000 bp flanking regions) that differentiate between modern sugar beet breeding lines from three seed companies and sugar beets from public seed banks; related to Table 3

Table S7. Genes containing variation specific for each company, along with functional information
