# Supplementary material for: Variation analysis using random forests reveals domestication patterns and breeding trends in sugar beet
Source: iScience. 2025 Jun 11;28(8):112835. doi: 10.1016/j.isci.2025.112835 (PMC12307672; doi:10.1016/j.isci.2025.112835)
Supplement: Document S1. Figures S1–S6 [file mmc1.pdf]

iScience, Volume *28*

Supplemental information

Variation analysis using random forests reveals domestication patterns and breeding trends in sugar beet

Felix L. Sandell, Christina Rupprecht, Heinz Himmelbauer, and Juliane C. Dohm

Variation analysis employing random forests reveals domestication patterns and breeding trends in sugar beet

Felix L. Sandell, Christina Rupprecht, Heinz Himmelbauer, Juliane C. Dohm

BOKU University, Vienna, Austria

Supplemental Figures

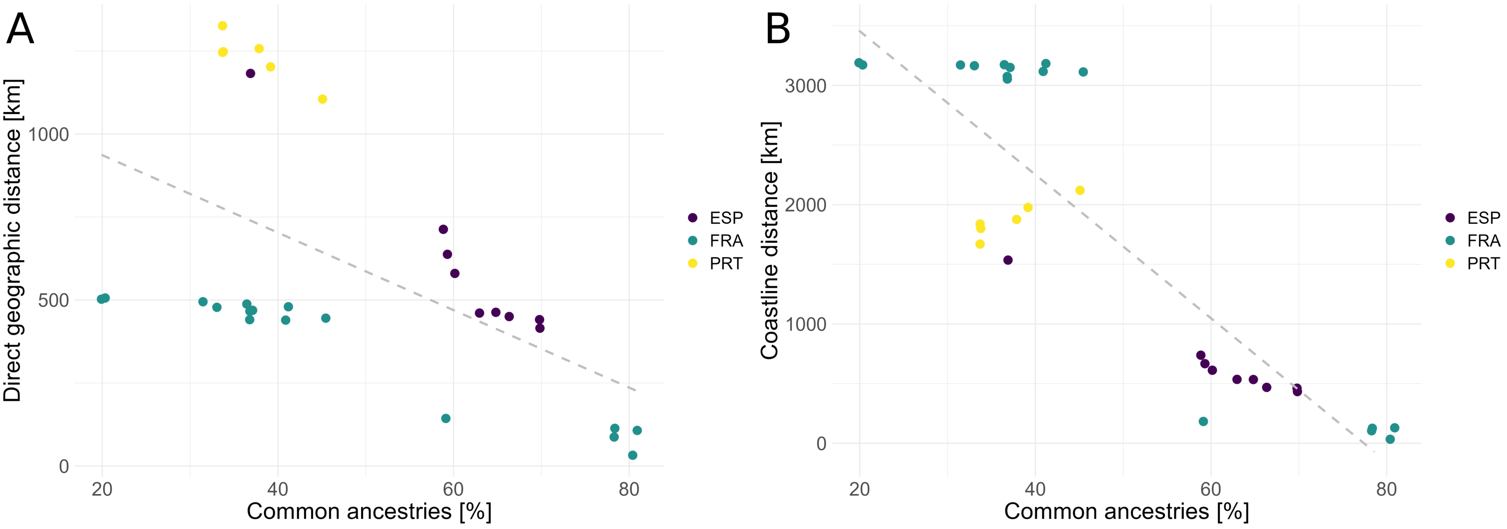

Figure S1: Correlation of genetic distance and linear distance (A) as well as coastline distance of width 1.2° (B) of *B. v. maritim*a accessions from France, Spain, and Portugal; related to Figure 1 and Table S2. Distances were plotted relative to accession PI 540563 from Southern France.

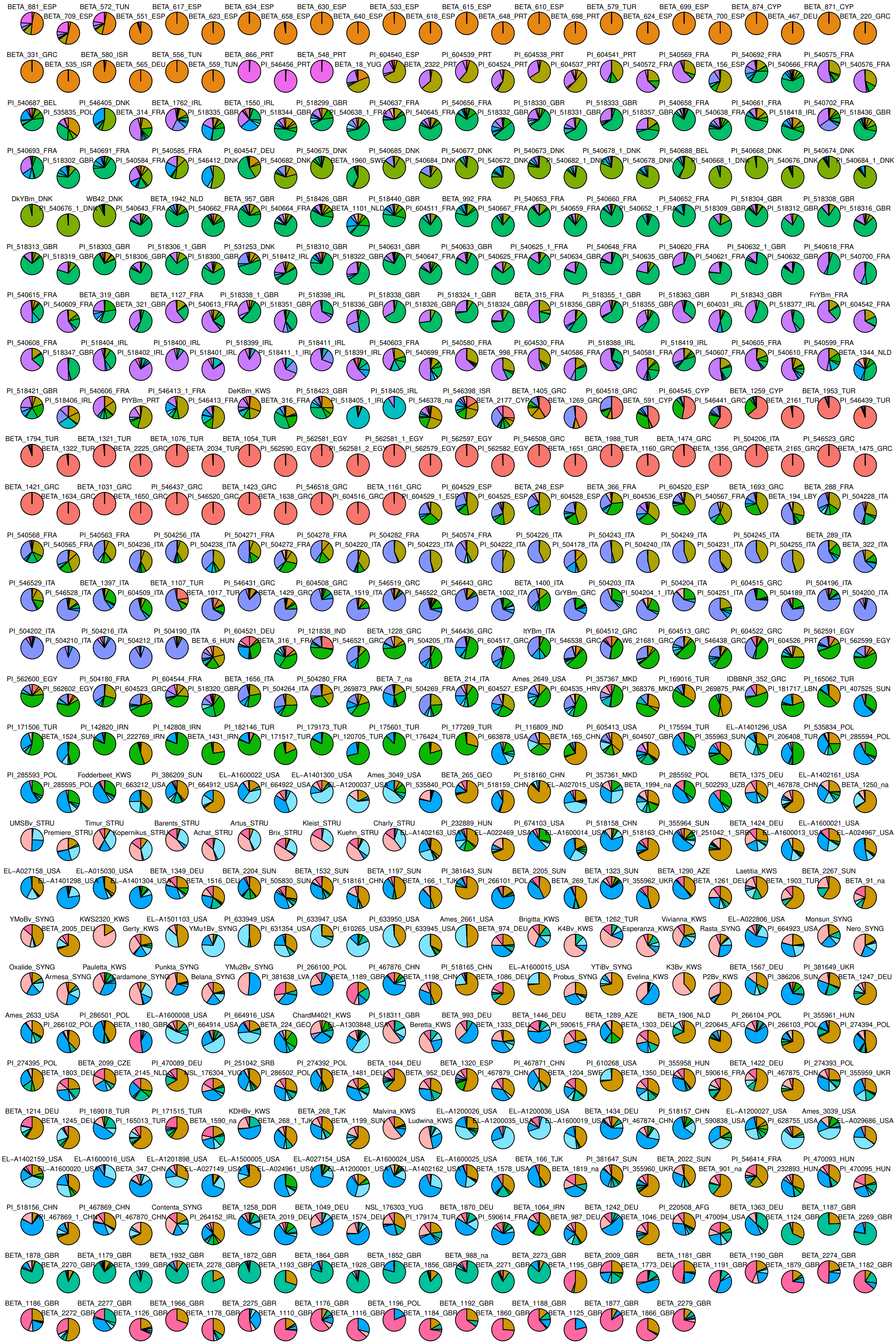
BETA_881_ESP
BETA_709_ESP
BETA_572_TUN
BETA_551_ESP
BETA_617_ESP
BETA_623_ESP
BETA_634_ESP
BETA_658_ESP
BETA_630_ESP
BETA_640_ESP
BETA_533_ESP
BETA_618_ESP
BETA_615_ESP
BETA_648_PRT
BETA_610_ESP
BETA_698_PRT
BETA_579_TUR
BETA_624_ESP
BETA_699_ESP
BETA_700_ESP
BETA_874_CYP
BETA_467_DEU
BETA_871_CYP
BETA_220_GRC
BETA_331_GRC
BETA_535_ISR
BETA_580_ISR
BETA_565_DEU
BETA_556_TUN
BETA_559_TUN
BETA_866_PRT
PI_546456_PRT
BETA_548_PRT
BETA_18_YUG
PI_604540_ESP
BETA_2322_PRT
PI_604539_PRT
PI_604524_PRT
PI_604538_PRT
PI_604537_PRT
PI_604541_PRT
PI_540572_FRA
PI_540569_FRA
BETA_156_ESP
PI_540692_FRA
PI_540666_FRA
PI_540575_FRA
PI_540576_FRA
PI_540687_BEL
PI_535835_POL
PI_546405_DNK
BETA_314_FRA
BETA_1762_IRL
PI_518335_GBR
BETA_1550_IRL
PI_518344_GBR
PI_518299_GBR
PI_540638_1_FRA
PI_540637_FRA
PI_540645_FRA
PI_540656_FRA
PI_518332_GBR
PI_518330_GBR
PI_518331_GBR
PI_518333_GBR
PI_518357_GBR
PI_540658_FRA
PI_540638_FRA
PI_540661_FRA
PI_518418_IRL
PI_540702_FRA
PI_518436_GBR
PI_540693_FRA
PI_518302_GBR
PI_540691_FRA
PI_540584_FRA
PI_540585_FRA
PI_546412_DNK
PI_604547_DEU
PI_540682_DNK
PI_540675_DNK
BETA_1960_SWE
PI_540685_DNK
PI_540684_DNK
PI_540677_DNK
PI_540672_DNK
PI_540673_DNK
PI_540682_1_DNK
PI_540678_1_DNK
PI_540678_DNK
PI_540688_BEL
PI_540668_1_DNK
PI_540668_DNK
PI_540676_DNK
PI_540674_DNK
PI_540684_1_DNK
DkYBm_DNK
PI_540676_1_DNK
WB42_DNK
PI_540643_FRA
BETA_1942_NLD
PI_540662_FRA
BETA_957_GBR
PI_540664_FRA
PI_518426_GBR
BETA_1101_NLD
PI_518440_GBR
PI_604511_FRA
BETA_992_FRA
PI_540667_FRA
PI_540653_FRA
PI_540659_FRA
PI_540660_FRA
PI_540652_1_FRA
PI_540652_FRA
PI_518309_GBR
PI_518304_GBR
PI_518312_GBR
PI_518308_GBR
PI_518316_GBR
PI_518313_GBR
PI_518319_GBR
PI_518303_GBR
PI_518306_GBR
PI_518306_1_GBR
PI_518300_GBR
PI_531253_DNK
PI_518412_IRL
PI_518310_GBR
PI_518322_GBR
PI_540631_GBR
PI_540647_FRA
PI_540633_GBR
PI_540625_FRA
PI_540625_1_FRA
PI_540634_GBR
PI_540648_FRA
PI_540635_GBR
PI_540620_FRA
PI_540621_FRA
PI_540632_1_GBR
PI_540632_GBR
PI_540618_FRA
PI_540700_FRA
PI_540615_FRA
PI_540609_FRA
BETA_319_GBR
BETA_321_GBR
BETA_1127_FRA
PI_540613_FRA
PI_518338_1_GBR
PI_518351_GBR
PI_518398_IRL
PI_518336_GBR
PI_518338_GBR
PI_518326_GBR
PI_518324_1_GBR
PI_518324_GBR
BETA_315_FRA
PI_518356_GBR
PI_518355_1_GBR
PI_518355_GBR
PI_518363_GBR
PI_604031_IRL
PI_518343_GBR
PI_518377_IRL
FrYBm_FRA
PI_604542_FRA
PI_540608_FRA
PI_518347_GBR
PI_518404_IRL
PI_518402_IRL
PI_518400_IRL
PI_518401_IRL
PI_518399_IRL
PI_518411_1_IRL
PI_518411_IRL
PI_518391_IRL
PI_540603_FRA
PI_540699_FRA
PI_540580_FRA
BETA_998_FRA
PI_604530_FRA
PI_540586_FRA
PI_518388_IRL
PI_540581_FRA
PI_518419_IRL
PI_540607_FRA
PI_540605_FRA
PI_540610_FRA
PI_540599_FRA
BETA_1344_NLD
PI_518421_GBR
PI_518406_IRL
PI_540606_FRA
PtYBm_PRT
PI_546413_1_FRA
PI_546413_FRA
DeKBm_KWS
BETA_316_FRA
PI_518423_GBR
PI_518405_1_IRL
PI_518405_IRL
PI_546378_na
PI_546398_ISR
BETA_2177_CYP
BETA_1405_GRC
BETA_1269_GRC
PI_604518_GRC
BETA_591_CYP
PI_604545_CYP
PI_546441_GRC
BETA_1259_CYP
BETA_2161_TUR
BETA_1953_TUR
PI_546439_TUR
BETA_1794_TUR
BETA_1322_TUR
BETA_1321_TUR
BETA_2225_GRC
BETA_1076_TUR
BETA_2034_TUR
BETA_1054_TUR
PI_562590_EGY
PI_562581_EGY
PI_562581_2_EGY
PI_562581_1_EGY
PI_562579_EGY
PI_562597_EGY
PI_562582_EGY
PI_546508_GRC
BETA_1651_GRC
BETA_1988_TUR
BETA_1160_GRC
BETA_1474_GRC
BETA_1356_GRC
PI_504206_ITA
BETA_2165_GRC
PI_546523_GRC
BETA_1475_GRC
BETA_1421_GRC
BETA_1634_GRC
BETA_1031_GRC
BETA_1650_GRC
PI_546437_GRC
PI_546520_GRC
BETA_1423_GRC
BETA_1638_GRC
PI_546518_GRC
PI_604516_GRC
BETA_1161_GRC
PI_604529_1_ESP
PI_604529_ESP
PI_604525_ESP
BETA_248_ESP
PI_604528_ESP
BETA_366_FRA
PI_604536_ESP
PI_604520_ESP
PI_540567_FRA
BETA_1693_GRC
BETA_194_LBY
BETA_288_FRA
PI_504228_ITA
PI_540568_FRA
PI_540565_FRA
PI_540563_FRA
PI_504236_ITA
PI_504256_ITA
PI_504238_ITA
PI_504271_FRA
PI_504272_FRA
PI_504278_FRA
PI_504220_ITA
PI_504282_FRA
PI_504223_ITA
PI_540574_FRA
PI_504222_ITA
PI_504226_ITA
PI_504178_ITA
PI_504243_ITA
PI_504240_ITA
PI_504249_ITA
PI_504231_ITA
PI_504245_ITA
PI_504255_ITA
BETA_289_ITA
BETA_322_ITA
PI_546529_ITA
PI_546528_ITA
BETA_1397_ITA
PI_604509_ITA
BETA_1107_TUR
BETA_1017_TUR
PI_546431_GRC
BETA_1429_GRC
PI_604508_GRC
BETA_1519_ITA
PI_546519_GRC
PI_546522_GRC
PI_546443_GRC
BETA_1002_ITA
BETA_1400_ITA
GrYBm_GRC
PI_504203_ITA
PI_504204_1_ITA
PI_504204_ITA
PI_504251_ITA
PI_604515_GRC
PI_504189_ITA
PI_504196_ITA
PI_504200_ITA
PI_504202_ITA
PI_504210_ITA
PI_504216_ITA
PI_504212_ITA
PI_504190_ITA
BETA_6_HUN
PI_604521_DEU
BETA_316_1_FRA
PI_121838_IND
PI_546521_GRC
BETA_1228_GRC
PI_504205_ITA
PI_546436_GRC
PI_604517_GRC
ItYBm_ITA
PI_546538_GRC
PI_604512_GRC
W6_21681_GRC
PI_604513_GRC
PI_546438_GRC
PI_604522_GRC
PI_604526_PRT
PI_562591_EGY
PI_562599_EGY
PI_562600_EGY
PI_562602_EGY
PI_504180_FRA
PI_604523_GRC
PI_604544_FRA
PI_518320_GBR
BETA_1656_ITA
PI_504264_ITA
PI_504280_FRA
PI_269873_PAK
BETA_7_na
PI_504269_FRA
BETA_214_ITA
PI_604527_ESP
Ames_2649_USA
PI_604535_HRV
PI_357367_MKD
PI_368376_MKD
PI_169016_TUR
PI_269875_PAK
IDBBNR_352_GRC
PI_181717_LBN
PI_165062_TUR
PI_407525_SUN
PI_171506_TUR
BETA_1524_SUN
PI_142820_IRN
PI_222769_IRN
PI_142808_IRN
BETA_1431_IRN
PI_182146_TUR
PI_171517_TUR
PI_179173_TUR
PI_120705_TUR
PI_175601_TUR
PI_176424_TUR
PI_177269_TUR
PI_663878_USA
PI_116809_IND
BETA_165_CHN
PI_605413_USA
PI_604507_GBR
PI_175594_TUR
PI_355963_SUN
EL-A1401296_USA
PI_206408_TUR
PI_535834_POL
PI_285594_POL
PI_285593_POL
PI_285595_POL
Fodderbeet_KWS
PI_663212_USA
PI_386209_SUN
PI_664912_USA
EL-A1600022_USA
PI_664922_USA
EL-A1401300_USA
EL-A1200037_USA
Ames_3049_USA
PI_535840_POL
BETA_265_GEO
PI_518159_CHN
PI_518160_CHN
EL-A027015_USA
PI_357361_MKD
BETA_1994_na
PI_285592_POL
PI_502293_UZB
BETA_1375_DEU
PI_467878_CHN
EL-A1402161_USA
BETA_1250_na
UMSBv_STRU
Premiere_STRU
Timur_STRU
Kopernikus_STRU
Barents_STRU
Achat_STRU
Artus_STRU
Brix_STRU
Kleist_STRU
Kuehn_STRU
Charly_STRU
EL-A1402163_USA
PI_232889_HUN
EL-A022469_USA
PI_674103_USA
EL-A1600014_USA
PI_518158_CHN
PI_518163_CHN
PI_355964_SUN
PI_251042_1_SRB
BETA_1424_DEU
EL-A1600013_USA
EL-A1600021_USA
EL-A024967_USA
EL-A027158_USA
EL-A1401298_USA
EL-A015030_USA
EL-A1401304_USA
BETA_1349_DEU
BETA_1516_DEU
BETA_2204_SUN
PI_505830_SUN
BETA_1532_SUN
PI_518161_CHN
BETA_1197_SUN
BETA_166_1_TJK
PI_381643_SUN
PI_266101_POL
BETA_2205_SUN
BETA_269_TJK
BETA_1323_SUN
PI_355962_UKR
BETA_1290_AZE
BETA_1261_DEU
Laetitia_KWS
BETA_1903_TUR
BETA_2267_SUN
BETA_91_na
YMoBv_SYNG
BETA_2005_DEU
KWS2320_KWS
Gerty_KWS
EL-A1501103_USA
YMu1Bv_SYNG
PI_633949_USA
PI_631354_USA
PI_633947_USA
PI_610265_USA
PI_633950_USA
PI_633945_USA
Ames_2661_USA
BETA_974_DEU
Brigitta_KWS
K4Bv_KWS
BETA_1262_TUR
Esperanza_KWS
Vivianna_KWS
Rasta_SYNG
EL-A022806_USA
PI_664923_USA
Monsun_SYNG
Nero_SYNG
Oxalide_SYNG
Armesa_SYNG
Pauletta_KWS
Cardamone_SYNG
Punkta_SYNG
Belana_SYNG
YMu2Bv_SYNG
PI_381638_LVA
PI_266100_POL
BETA_1189_GBR
PI_467876_CHN
BETA_1198_CHN
PI_518165_CHN
BETA_1086_DEU
EL-A1600015_USA
Probus_SYNG
YTiBv_SYNG
Evelina_KWS
K3Bv_KWS
P2Bv_KWS
BETA_1567_DEU
PI_386206_SUN
PI_381649_UKR
BETA_1247_DEU
Ames_2633_USA
PI_266102_POL
PI_286501_POL
BETA_1180_GBR
EL-A1600008_USA
PI_664914_USA
PI_664916_USA
BETA_224_GEO
ChardM4021_KWS
EL-A1303848_USA
PI_518311_GBR
Beretta_KWS
BETA_993_DEU
BETA_1333_DEU
BETA_1446_DEU
PI_590615_FRA
BETA_1289_AZE
BETA_1303_DEU
BETA_1906_NLD
PI_220645_AFG
PI_266104_POL
PI_266103_POL
PI_355961_HUN
PI_274394_POL
PI_274395_POL
BETA_1803_DEU
BETA_2099_CZE
BETA_2145_NLD
PI_470089_DEU
NSL_176304_YUG
PI_251042_SRB
PI_286502_POL
PI_274392_POL
BETA_1481_DEU
BETA_1044_DEU
BETA_952_DEU
BETA_1320_ESP
PI_467879_CHN
PI_467871_CHN
BETA_1204_SWE
PI_610268_USA
BETA_1350_DEU
PI_355958_HUN
PI_590616_FRA
BETA_1422_DEU
PI_467875_CHN
PI_274393_POL
PI_355959_UKR
BETA_1214_DEU
BETA_1245_DEU
PI_169018_TUR
PI_165013_TUR
PI_171515_TUR
BETA_1590_na
KDHBv_KWS
BETA_268_1_TJK
BETA_268_TJK
BETA_1199_SUN
Malvina_KWS
Ludwina_KWS
EL-A1200026_USA
EL-A1200035_USA
EL-A1200036_USA
EL-A1600019_USA
BETA_1434_DEU
PI_467874_CHN
PI_518157_CHN
PI_590838_USA
EL-A1200027_USA
PI_628755_USA
Ames_3039_USA
EL-A029686_USA
EL-A1402159_USA
EL-A1600020_USA
EL-A1600016_USA
BETA_347_CHN
EL-A1201898_USA
EL-A027149_USA
EL-A1500005_USA
EL-A024961_USA
EL-A027154_USA
EL-A1200001_USA
EL-A1600024_USA
EL-A1402162_USA
EL-A1600025_USA
BETA_1578_USA
BETA_166_TJK
BETA_1819_na
PI_381647_SUN
PI_355960_UKR
BETA_2022_SUN
BETA_901_na
PI_546414_FRA
PI_232893_HUN
PI_470093_HUN
PI_470095_HUN
PI_518156_CHN
PI_467869_1_CHN
PI_467869_CHN
PI_467870_CHN
Contenta_SYNG
PI_264152_IRL
BETA_1258_DDR
BETA_2019_DEU
BETA_1049_DEU
BETA_1574_DEU
NSL_176303_YUG
PI_179174_TUR
BETA_1870_DEU
PI_590614_FRA
BETA_1064_IRN
BETA_987_DEU
BETA_1242_DEU
BETA_1046_DEU
PI_220508_AFG
PI_470094_USA
BETA_1363_DEU
BETA_1124_GBR
BETA_1187_GBR
BETA_2269_GBR
BETA_1878_GBR
BETA_2270_GBR
BETA_1179_GBR
BETA_1399_GBR
BETA_1932_GBR
BETA_2278_GBR
BETA_1872_GBR
BETA_1193_GBR
BETA_1864_GBR
BETA_1928_GBR
BETA_1852_GBR
BETA_1856_GBR
BETA_988_na
BETA_2271_GBR
BETA_2273_GBR
BETA_1195_GBR
BETA_2009_GBR
BETA_1773_DEU
BETA_1181_GBR
BETA_1191_GBR
BETA_1190_GBR
BETA_1879_GBR
BETA_2274_GBR
BETA_1182_GBR
BETA_1186_GBR
BETA_2272_GBR
BETA_2277_GBR
BETA_1126_GBR
BETA_1966_GBR
BETA_1178_GBR
BETA_2275_GBR
BETA_1110_GBR
BETA_1176_GBR
BETA_1116_GBR
BETA_1196_POL
BETA_1184_GBR
BETA_1192_GBR
BETA_1860_GBR
BETA_1188_GBR
BETA_1125_GBR
BETA_1877_GBR
BETA_1866_GBR
BETA_2279_GBR

Figure S2 (previous page): Admixture pie charts for 667 *Beta* accessions ordered by the phylogenetic tree based on intergenic variants from Felkel et al. [1] re-rooted at BETA 881; related to Figures 2 and 3. Color legend see Figure S3.

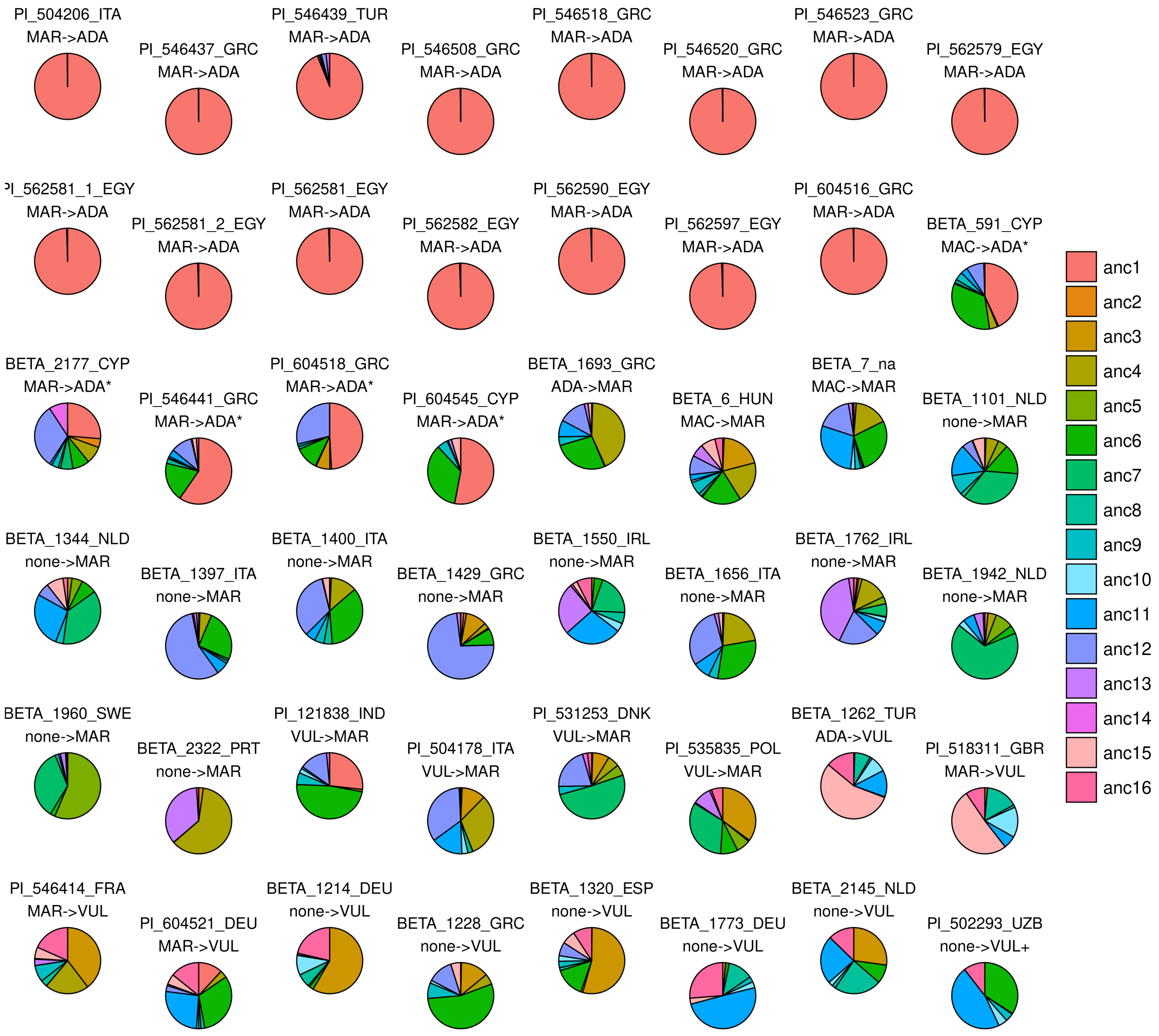

Figure S3: Admixture pie charts for previously re-classified accessions [2]; related to Figures 2 and 3. Some of the suggested re-assignments were not clearly supported by admixture proportions. ADA: *B. v. adanensis*, MAC: *B. macrocarpa*, MAR: *B. v. maritima*, VUL: *B. v. vulgaris*, none: previously "*Beta* sp.". Example: "MAR -> ADA" means that the accession was "MAR" according to the seed bank but was re-classfied to "ADA" by Sandell et al. [2].

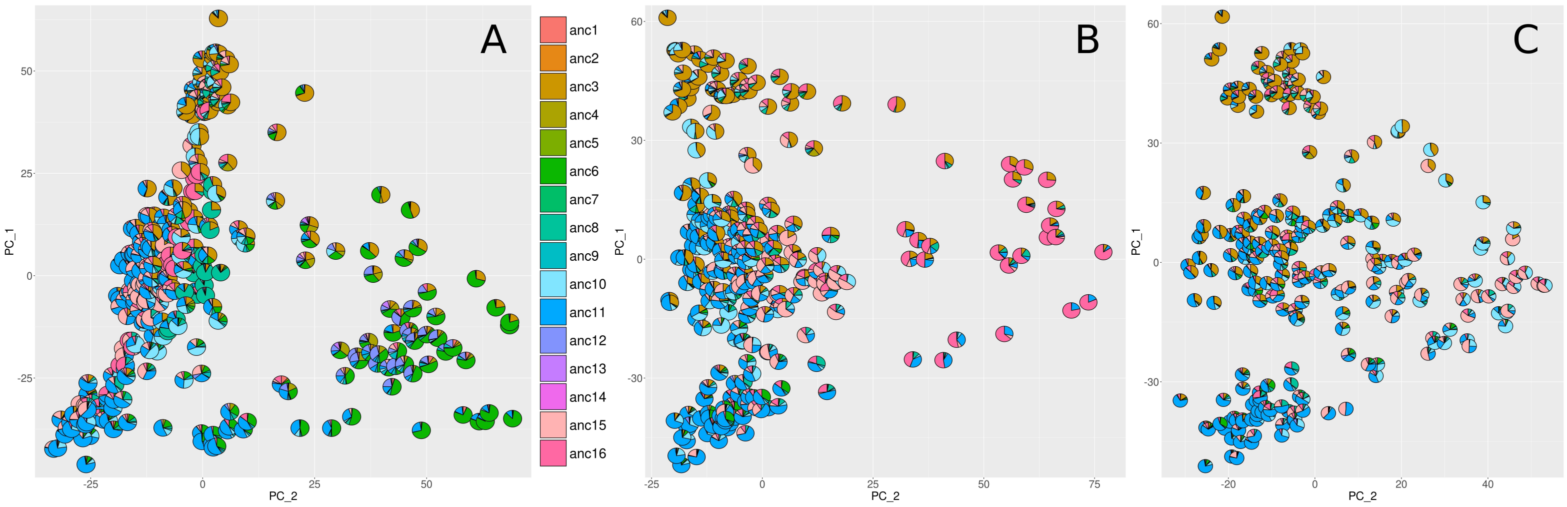

Figure S4: PCA plots based on admixture proportions of accessions containing "ancestry 6" (green) and all sugar beet accessions (A), of sugar beet accessions after removal of accessions dominated by "ancestry 8" (B), and after removal of sugar beet accessions dominated by "ancestry 8" or "ancestry 16" (C); related to Figure 3.

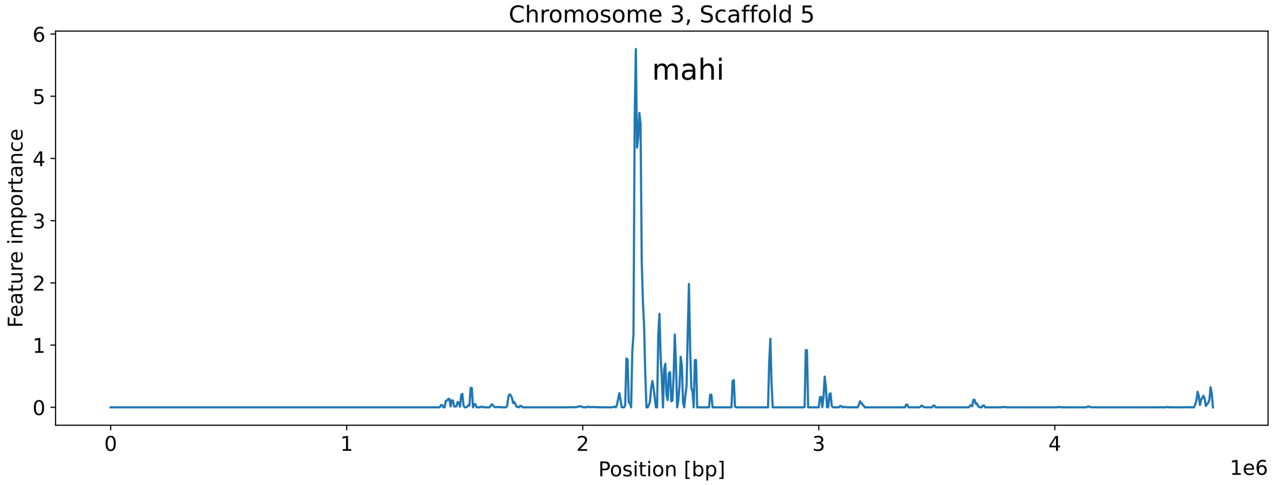

Figure S5: Graphical display of the feature importance peak belonging to variants at the locus of the *Erysiphe* resistance candidate gene, selected for by all three breeding companies; related to Table 3.

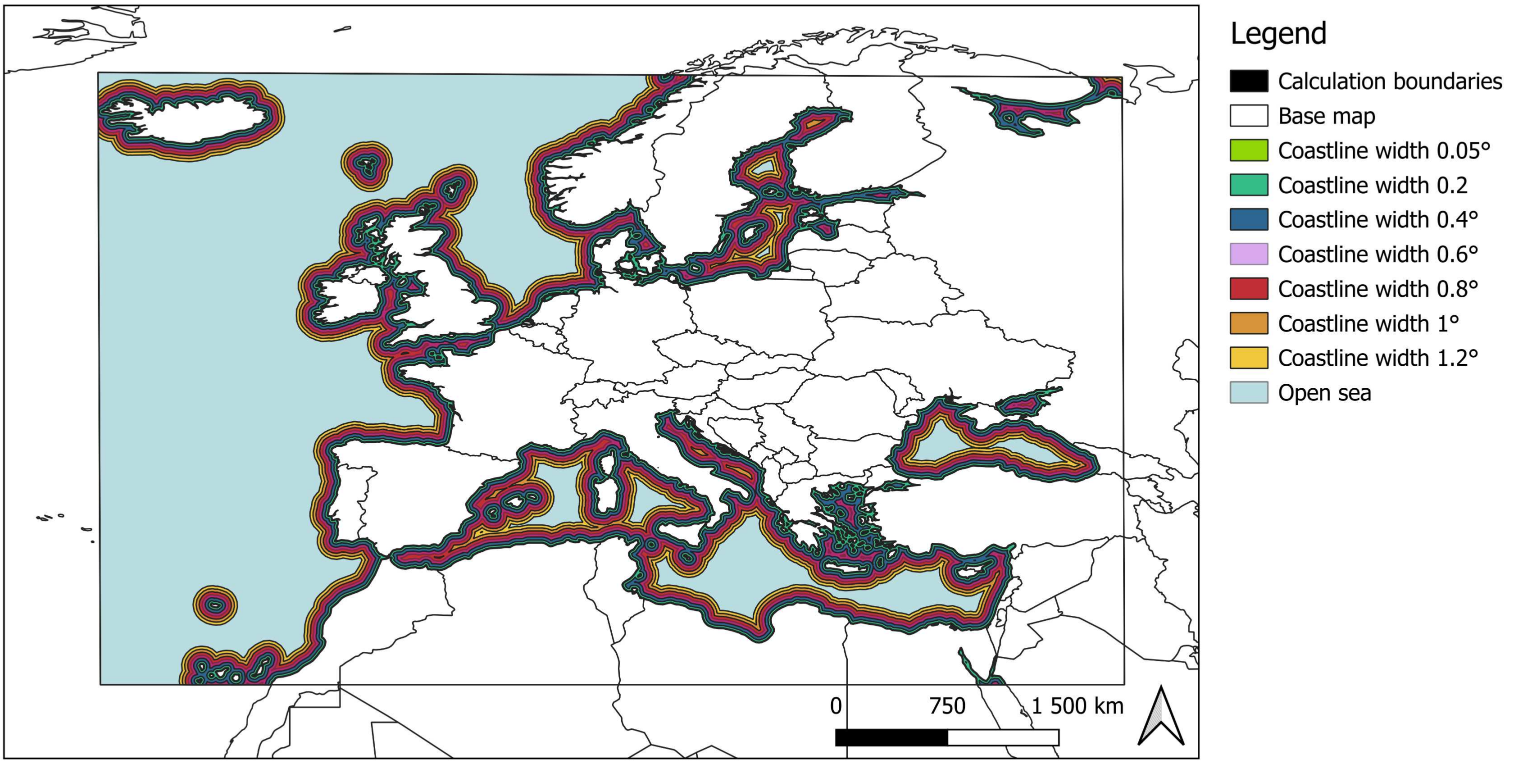

Figure S6: Illustration of different coastline width definitions; related to STAR Methods. One degree corresponds to about 111 km.

References

1. Felkel, S., Dohm, J.C., and Himmelbauer, H. (2023). Genomic variation in the genus Beta based on 656 sequenced beet genomes. Sci. Rep. *13*, 8654. https://doi.org/10.1038/s41598-023-35691-7.
2. Sandell, F.L., Stralis-Pavese, N., McGrath, J.M., Schulz, B., Himmelbauer, H., and Dohm, J.C. (2022). Genomic distances reveal relationships of wild and cultivated beets. Nat. Commun. *13*, 2021. https://doi.org/10.1038/s41467-022-29676-9.
